# Mutational Screening of LCA Genes Emphasizing *RPE65* in South Indian Cohort of Patients

**DOI:** 10.1371/journal.pone.0073172

**Published:** 2013-09-16

**Authors:** Anshuman Verma, Vijayalakshmi Perumalsamy, Shashikant Shetty, Maigi Kulm, Periasamy Sundaresan

**Affiliations:** 1 Department of Genetics, Dr. G. Venkataswamy Eye Research Institute, Aravind Medical Research Foundation, Aravind Eye Hospital, Madurai, Tamil Nadu, India; 2 Pediatric Eye Clinic, Aravind Eye Hospital, Madurai, Tamil Nadu, India; 3 Asper Biotech, Tartu, Estonia; Innsbruck Medical University, Austria

## Abstract

**Background:**

Leber congenital amaurosis (LCA) is the most severe form of inherited retinal visual impairment in children. So far, mutations in more than 20 genes have been known to cause LCA and among them, *RPE65* is a suitable candidate for gene therapy. The mutational screenings of *RPE65* and other LCA genes are requisite in support of emerging gene specific therapy for LCA. Therefore, we have carried out a comprehensive LCA genes screening using a combined approach of direct sequencing and DNA microarray based Asper chip analysis.

**Methodology/Principal Findings:**

Thirty clinically diagnosed index LCA cases from Southern India were screened for coding and flanking intronic regions of *RPE65* through direct sequencing. Among thirty, 25 cases excluded from *RPE65* mutations were subjected to Asper chip analysis, testing 784 known pathogenic variations in 15 major LCA genes. In *RPE65* screening, four different pathogenic variations including two novel (c.361insT & c.939T>A) and two known (c.394G>A & c.361delT) mutations were identified in five index cases. In the chip analysis, seven known pathogenic mutations were identified in six index cases, involving genes *GUCY2D, RPGRIP1, AIPL1, CRX and IQCB1*. Overall, 11 out of 30 LCA cases (36.6%) revealed pathogenic variations with the involvement of *RPE65* (16.6%), *GUCY2D* (10%), *RPGRIP1* (3.3%), *AIPL1* (3.3%) and *CRX* & *IQCB1* (3.3%).

**Conclusions/Significance:**

Our study suggests that such combined screening approach is productive and cost-effective for mutation detection and can be applied in Indian LCA cohort for molecular diagnosis and genetic counselling.

## Background

Leber congenital amaurosis (LCA; [OMIM] #204000) is a congenital retinal disorder causing severe visual impairment before the age of two years [1]. Though, usually considered as a rare disorder with reported prevalence of 2 to 3 per 100,000 live births,[2] its incidence appears to be higher in consanguineous population like South India [3], [4]. Clinically LCA is a pool of different phenotypes. The severe vision loss is accompanied with sensory nystagmus, sluggish eye pupil responses, high refractive errors, heterogeneous retinal changes and reduced or absent electroretinogram (ERG) responses. The other features which may or may not be present includes pressing and poking of eyes, photoaversion, nyctalopia, keratoconus and cataract [5]. LCA is caused by mutations in 23 genes meanwhile; the major contributing one includes *GUCY2D, CRB1, RPE65, CEP290, RPGRIP1, RDH12, AIPL1, CRX, LCA5, TULP1, LRAT, IMPDH1, RD3, MERTK, SPATA7* [6]. These genes all together account for 70% of LCA cases, and their individual mutational frequency may vary from one ethnic population to other [5], [6], [7]. Depending on the underlying gene mutations, some LCA phenotypes may be absent or prominent or may arise in later infancy of life [8]. LCA is typically inherited as an autosomal recessive form, although few mutations show the autosomal dominant pattern of inheritance too [9], [10]. For the management of LCA patients, the most promising aspect is the gene therapy success for RPE65 LCA cases, achieved in phase 2&1 clinical trials and previous animal model studies, which have paved the pathway to cure inherited blindness [11], [12]. The animal model based gene therapy trials for other LCA genes like LCA1 (*GUCY2D*), LCA4 (*AIPL1*) and LCA6 (*RPGRIP1*) are also in progress, which further provides a prospect for the effective treatment regime in inherited blinding retinal disorders [13], [14], [15]. Since, future treatment will be geared towards gene specific therapies, studies involving mutational screening of LCA genes, particularly *RPE65* are prerequisites to establish a pool of patients who could be benefited by such emerging treatments. Among the available gene screening system, the DNA micro-array based Asper LCA chip (http://www.asperophthalmics.com/Leber congenital amaurosis DNA test) offers a rapid, cost-effective and accurate genotyping method for detection of all known variations associated with LCA [16], [17]. Though, the chip is limited to detect only known variations, studies show that in approximately 60–70% of cases, at least one disease-associated mutation could be identified using this technique [18]. The chip applies allele-specific primer extension analysis and can be repeatedly updated with new mutations published. Although, many studies have implemented the LCA chip array, it has not been executed in South Indian cohort of LCA patients. Screening strategy combining advance mutation detection methods with automated DNA sequencing can increase the efficiency and efficacy of mutation detection. Therefore, we applied a combined approach, where direct DNA sequencing of *RPE65* gene was followed by an Asper chip analysis, among cases excluded from *RPE65* mutations. In addition, in silico characterization for the identified mutations and related clinical phenotype of patients were studied.

## Materials and Methods

### LCA Patients

Thirty unrelated clinically diagnosed LCA patients with mean age of 4 years of early diagnosis, including 19 male and 11 female were recruited from the pediatric eye clinic, Aravind eye hospital, Madurai, between the period of 2008 to 2011. Among thirty, 27 probands were born through consanguineous marriage. The onset of disease was since birth in all LCA patients. The diagnosis was based on clinical characteristics of LCA, confirmed with undetectable or significantly reduced ERG. Patients with a late onset of disease after five years, and associated with any syndromic features like mental retardation or other systemic failures were excluded from the study. Hundred unrelated ethnic matched healthy blood donors were used as a control panel. All subjects belonged to South Indian ethnicity involving the states of Tamil Nadu, Kerala and Andhra Pradesh.

### Ethics

The written informed consents were received from all participants and for the minor subjects, it was obtained from their parents or legal guardians. The pedigrees were constructed for each probands, based on the information provided by guardians. The study obeyed to the tenets of the declaration of Helsinki and was approved by the institutional review board, Aravind eye hospital, Madurai, Tamil Nadu, India. The blood samples were collected from all study subjects. The genomic DNA was extracted from the peripheral blood samples using modified salt precipitation method [19]. For the microarray based chip analysis, DNA samples were exported to Asper biotech, Estonia (http://www.asperbio.com). The transfer of genomic DNA followed the memorandum of ministry of Health & F.W. India, and was permitted by the Indian council of medical research (ICMR), New Delhi, India.

### RPE65 Mutational Screening

Thirty LCA patients underwent sequencing analysis of *RPE65*. Fourteen exons of *RPE65* with their flanking boundaries were amplified from the genomic DNA by 13 sets of primers (exon7&8 combined) designed with Primer3 and Primer BLAST software tools. PCR was done in 80 µl of reaction set-up with respective exons annealing temperature at 59°C (exon1), 51°C (exon4), 55°C (exon 2,3,6,9,11,12,14) and touch down at 55°C (exon 5,7&8,10,13). The amplicons were run in 1% agarose gel and purified using the gel-elution kit method (Bio Basic Inc. Canada). The purified amplicons were sequenced using Big Dye Terminator ready reaction mix and analyzed on an ABI-3130 genetic analyzer (Applied Biosystems, Fostercity, CA). The sequence analysis was done using BLAST software and compared with the Ensemble nucleotide sequence of *RPE65* gene [Gen Bank accession number NM_000329.2].

### LCA chip analysis

After direct sequencing analysis, twenty-five index cases excluded from *RPE65* mutations were subjected to APEX chip screening. The Asper chip was updated by the collaborating authors and designed to contain all known LCA pathogenic variants, including Asian specific variations. Database used for reference variations included Online Mendelian Inheritance in Man (OMIM); http://www.ncbi.nlm.nih.gov/omim, Retina International Scientific Newsletter Mutation Database; http://www.retina-international.org and RetNet Retinal Information Network; https://sph.uth.edu/retnet/. In the updated version (LCA microarray version –9.0), a total of 784 known variations were probed from 15 LCA genes including *AIPL1* [NM_014336.3], *CRB1* [NM_201253.2], *CRX* [NM_000554.4], *GUCY2D* [NM_000180.3], *LRAT* [NM_004744.3], *MERTK* [NM_006343.2], *CEP290* [NM_025114.3], *RDH12* [NM_152443.2], *RPGRIP*1 [NM_020366.3], *RPE65* [NM_000329.2], *TULP1* [NM_003322.3], *LCA5* [NM_001122769.2], *SPATA7* [NM_018418.4], *IQCB1* [NM_001023570.2], *RD3* [NM_001164688.1].

The analysis was done based on the prescribed protocol described elsewhere (www.asperbio.com; Asper Biotech, Ltd.) [20], [21]. Each of the mutations detected in the LCA chip analysis was subsequently confirmed through direct sequencing.

### Bioinformatics assessment

The primary evaluation of identified variations was done using Alamut software version 2.1e (Interactive Biosoftware, Rouen, France) aided with tools like PolyPhen (Polymorphism Phenotyping) and SIFT (Sorting Intolerant From Tolerant). The nomenclature, location and classification of variations were based on Alamut output. In addition, published data informations and other bioinformatic tools or prediction programs available through the ExPASy World Wide Web (www.expasy.org) server were used for the characterization of mutations. Each mutation's position was looked for domain, conserved region and stability of the encoded protein.

### Clinical evaluation

The patients with identified mutations were reviewed and clinically evaluated at the hospital. Each patient was examined for diagnostic primary and secondary features of LCA. The clinical re-evaluation involved assessment of visual status, (best corrected visual acuity according to Snellen charts), retinoscopy, axial length measurement, keratometry (K-reading), slit lamp evaluation and indirect ophthalmoscopy. The fundus photograph using the retinal camera (TOPCON TRC-50 DX, Japan) and foveal thickness using optical coherence tomography (3D OCT; Topcon, Tokyo, Japan) were examined in co-operative patients. The scotopic and photopic visual electroretinogram were performed for each LCA case previously during their clinical examination under general anaesthesia with ERG system (Kurbisfeld, UTAS 3000, LKC Technologies) using corneal contact lens electrodes. The ERG protocol complied with the standards published by the International society for clinical electrophysiology of vision (ISCEV).

## Results

The combined approach of *RPE65* direct sequencing and Asper chip analysis revealed ten different disease causing variations in 6 LCA genes from 11 LCA patients (**Table 1**). Two cases carried the same *RPE65* mutation, and another two cases contained the same *GUCY2D* mutation. All mutations were confirmed in patients and checked in their unaffected parents by direct sequencing. The available healthy parents were found to be heterozygous for the mutations detected in their affected children. The identified mutations in direct sequencing analysis were absent in 100 controls, while 50 controls were excluded for the identified known mutations in Asper chip analysis. The clinical findings of patients confirmed with mutations were evaluated (**Table 2**). In addition, few reported polymorphisms including two putative variations were detected among LCA patients (**Table 3**).

**Table 1:** Summary of the identified pathogenic mutations in LCA patients in this study.

S.No.	Patient ID	Gene	Reference ID	Identified Variations	Amino Acid Change	SIFT score	Clinical Significance
**1**	LCA44–1	RPE65	NM_000329.2	HOM	p.S121F	NA	Pathogenic
				c.361insT	FS*10		
				(Novel)			
**2**	LCA74–1	RPE65	NM_000329.2	HOM	p.H313Q	0.29	Pathogenic
				c.939T>A		(Tolerated)	
				(Novel)			
**3**	LCA51–1	RPE65	NM_000329.2	HOM	p.S121L	NA	Pathogenic
				c.361delT	FS*6		
**4**	LCA72–1	RPE65	NM_000329.2	HOM	p.S121L	NA	Pathogenic
				c.361delT	FS*6		
**5**	LCA 68–1	RPE65	NM_000329.2	HET	p.A132T	0.11	Mild Pathogenic
				c.394G>A		(Tolerated)	
**6**	LCA55–1	GUCY2D	NM_000180.3	HOM	p.R1040G	0.00	Pathogenic
				c.3118C>G		(Deleterious)	
**7**	LCA–87–1	GUCY2D	NM_000180.3	HOM	p.R1040G	0.00	Pathogenic
				c.3118C>G		(Deleterious)	
**8**	LCA 84–1	GUCY2D	NM_000180.3	HOM	p.S1023_V	NA	Pathogenic
				c.3068_3069ins	1025dup		
				CACTGTGAG			
				(Novel)			
**9.**	LCA69–1	AIPL1	NM_014336.3	HOM	p.W278X	NA	Pathogenic
				c.834G>A			
**10**	LCA53–1	CRX	NM_000554.4	HET c.196G>A	p.V66I	0.00	Mild pathogenic
		IQCB1	NM_001023570.2	HOM c.1465C>T	p.R489X	(Deleterious)	Pathogenic
						NA	
**11.**	LCA81–1	RPGRIP1	NM_020366.3	HOM	p.R1189X	NA	Pathogenic
				c.3565C>T			

**S.No.1**–**5:** Mutations identified by direct sequencing analysis of *RPE65*.

**S.No.6**–**11:** Mutations identified in Asper chip analysis and confirmed by direction sequencing.

Nucleotide numbering represents cDNA numbering with +1 corresponding at the start of the coding sequence (i.e. from the first A of the translation initiation codon, ATG) in the reference sequence according to nomenclature guidelines (www.hgvs.org/mutnomen).

RPE65 c.361del T mutation was identified in two index LCA cases (LCA51–1 and LCA72–1).

GUCY2D c.3118 C>G mutation was identified in two index LCA cases (LCA55–1 and LCA87–1).

Patient LCA68–1 was found to be with single heterozygous RPE65 mutation which alone may not be conclusive to explain the LCA phenotype.

Patient LCA53–1 carried heterozygous CRX mutation along with homozygous IQCB1 mutation; both together can sufficiently explain the LCA phenotype.

SIFT score ranges from 0 to 1; value between 0.00–0.05 is considered deleterious.

**Abbreviation:** HOM- Homozygous, HET- Heterozygous, FS- Frame Shift, NA-Not applicable.

**Table 2:** Summary of the clinical features of LCA patients identified with mutations in this study.

11	10	9	8	7	6	5	4	3	2		1	S.NO		
LCA81–1	LCA69–1	LCA53–1	LCA84–1	LCA87–1	LCA55–1	LCA74–1	LCA72–1	LCA68–1	LCA51–1		LCA44–1	Patient ID		
3Y	5 Y	7 Y	2 Y	11 Y	4 Y	4 Y	6 Y	4 Y	7 Y		3 Y	Age @ ecent followup		
F	M	M	M	F	M	F	F	M	M		M	Gender		
Yes	Yes	Yes	No	Yes	Yes	Yes	Yes	No	Yes		No	Consan uinity		
Vertical Pendular	Rotatory	Horizontal Pendular	Horizontal jerky	Horizontal Jerky	Biplanar Pendular	Asymmetrical Pendular, RE>LE	Latent Jerky	Asymmetrical Pendular, RE>LE	Absent	Pendular RE>LE	Asymmetrical	Nystagmus		
NA	NA	3/60	NA	4/60	6/60	4/60	6/60	NA	6/19		4/60	RE	Visual acuity	
NA	NA	4/60	NA	4/60	6/60	4/60	5/60	NA	6/19		4/60	LE		
_+_5.5	_+_3.5	−3.0 * 180	_+_7.0	_+_4.0	−1.0*180	_+_9.0	−1.05* 180	+1.50	+2.4		+3.0	RE	Refractive error	
_+_5.5	_+_3.5	−3.0*180	_+_8.0	_+_4.0	−1.0*180	_+_7.0	−2.5*180	+1.0	+2.4		+2.5	LE		
21.07	21.34	22.92	18.81	21.17	NA	20.42	NA	21.94	NA		NA	RE	Axial length (mm)	
21.30	21.43	23.22	18.85	21.28	NA	20.49	NA	21.72	NA		NA	LE		
36.0	44.0	44.25	NA	44.75	43.50	47.0	NA	42.50	NA		NA	K1	RE	K –reading (D)
37.0	43.0	47.25	NA	42.50	42.50	49.0	NA	45.75	NA		NA	K2		
35.25	43.50	44.50	NA	45.75	43.00	44.25	NA	44.0	NA		NA	K1	LE	
35.50	43.00	47.50	NA	43.0	41.75	46.25	NA	46.0	NA		NA	K2		
Oculodigital sign	Nil	Photophobia	Photophobia	Nil	Photophobia	Night blindness	Alternate exotropia	Photophobia Night blindness	Night blindness		Nil	Secondary features		
Normal disk PE mottling	Waxy disc pallor, Severe RPE mottling, vessel attenuation, Foveal reflex absent	Disc – mild pallor, Mild arteriolar attenuation ine RPE mottling in the periphery	Within normal limits	Normal disk ild arterial attenuation	Within normal limits	Vascular attenuation, Disc pallor, oveal reflex absent	RPE mottling, Vascular attenuation, Salt & pepper pigmentation, Vascular attenuation	Within normal limits	Normal disk, Mild vessel attenuation, PE mottling, White dot in the periphery		Vascular attenuaton, White pigmentation in the mid periphery	Fundus ppearance		
NC	Loss of foveal thickness RE-63µ; LE-31µJunctional complex disruption, Disc edema	Normal Foveal thickness E-175µ, LE-158µ	NC	Normal Foveal thickness E-155µ; LE-131µ	NC	Loss of foveal thickness E-115µ; LE-95µ	NC	NC	Loss of foveal thickness E -103µ; RE -102µ		NC	OCT		

Abbreviation: Y- Year, M- Male, F- Female, RE- Right Eye, LE- Left Eye, K reading- Keratometry reading, D- Diaptore OCT- Optical coherence tomography, NA – Not available, NC- Not Co-operated.

LCA81-1 has K1, K2 reading <43D in both RE & LE, suggesting a cornea plana.

LCA72–1 bears alternate exotropia as a secondary feature.

**Table 3:** Identified polymorphisms in this study among LCA cases.

Gene	Polymorphism	Exon/Intron position	Amino acid change	rs No.	Frequency in cases
RPE65	c.1056 G>A	Ex-10	p.E352E	rs12145904	11/30
RPE65	c.1338+20A>C	Int-7		rs12564647	1/25
*RPE65	c.868C>T	Ex-9	p.H290Y		3/30
AIPL1	c.268G>C	Ex-2	p.D90H	rs12449580	3/25
AIPL1	c.286G>A	Ex-3	p. V96I	rs62619924	6/25
GUCY2D	c.61T>C	Ex-2	p.W21R	rs9905402	4/25
GUCY2D	c.154G>T	Ex-2	p.A52S	rs61749665	20/25
GUCY2D	c.2101C>T	Ex-10	p. P701S	rs34598902	5/25
GUCY2D	c.2345T>A	Ex-12	p.L782H	rs8069344	3/25
RPGRIP1	c.574A>G	Ex-4	p.K192E	rs6571751	19/25
RPGRIP1	c.907-17 del TAA	Int-6			14/25
RPGRIP1	c.3097 G>C	Ex-18	p.E1033Q	rs3748361	19/25
*RPGRIP1	c.3560_3566 del 7bp	Ex-22	p.G1188R_1189delFS		1/25
≠RPGRIP1	c.1639G>T	Ex-13	p.A547S	rs10151259	5/25

The above listed polymorphisms were identified among LCA cases during direct sequencing as well as Asper chip analysis.

* c.868C>T in *RPE65* and c.3560_3566 Del 7bp in *RPGRIP1* were observed as a putative change.

≠ *RPGRIP1* c.1639G>T was found as a non-pathogenic change.

### *RPE65* screening

In the direct sequencing analysis of *RPE65*, in five LCA patients, four different pathogenic mutations, which include two novel and two reported mutations were identified. A novel homozygous *RPE65* thymine insertional mutation c.361insT was identified in one patient (LCA44–1). The mutation lies in the highly conserved carotenoid oxygenase domain and creates a frame shift, which leads to a stop codon nine positions downstream, resulting in a truncated *RPE65* protein of 129 amino acids. The mutation was submitted to dbSNP data base with assigned SNP ID [**rs121918844**]. Another novel homozygous *RPE65* mutation c.939 T>A (p.H313Q) was identified in patient (LCA74–1). His313 is one of the highly conserved residues across eighteen species in the carotenoid oxygenases domain of *RPE65*. The crystal structure of *RPE65* shows that iron-ion acts as a cofactor and is directly coordinated by the imidazole nitrogen atom (N-atoms) of four histidine residues His180, His241, His527 and His313, with average bond length of 2.2 Å. Functionally, It was demonstrated that mutation in any one of these His residues completely abolishes the enzymatic activity of *RPE65*, suggesting that these conserved residues are essential for its isomerohydrolase activity [22]. The protein stability prediction tools like MuPro and FoldX also showed a decrease in the stability due to this substitution. A homozygous deletion of thymine nucleotide at c.361 position of *RPE65* was observed in two LCA patients (LCA51–1& LCA72–1). The mutation was previously observed in Portugal cohort of LCA patients as a de novo mutation [7] as well as reported in Indian LCA patients [23]. It lies in the highly conserved Ser121 carotenoid oxygenases domain of *RPE65* and creates a codon frame shift, which generates a stop codon five positions downstream, forming a truncated protein of 125 amino acids. A heterozygous c.394G>A (p.A132T) *RPE65* mutation was seen in one patient (LCA68–1). The parental DNA analysis showed father as heterozygous while mother was normal for this variation. This variant has been previously reported in RP patients in homozygous [24] as well as in heterozygous form [25], [26]. The alanine 132 amino acid position is moderately conserved. The protein stability prediction tools indicated a reduction in protein stability. In vitro functional study showed a 50% decrease in the isomerohydrolase activity caused due to this mutation [27]. The occurrence of this variant in unaffected father and retention of 50% wild type activity in a biochemical study suggests its mild pathogenicity. However, being in a heterozygous state, this change alone may not be sufficient to explain the disease phenotype and the patient might have an additional change which together leads to the pathogenesis of the disease.

### LCA chip

In the LCA chip analysis, 6 of 25 LCA patients revealed potentially disease causing variations in genes *GUCY2D, AIPL1, RPGRIP1, CRX* and *IQCB1*. A homozygous missense mutation *GUCY2D* c.3118C>G (p.R1040G) was identified in two patients (LCA55–1 & LCA87–1). The mutation lies at the conserved carboxy-terminal adenylyl cyclase (guanylyl cyclase) domain of the protein. MuPro and FoldX, found a decrease in the stability of protein caused due to this mutation. In one LCA case (LCA84–1), an indel of 7 bp deletion with 2bp insertion in *GUCY2D* gene was predicted by Asper chip analysis. However, after confirmation with direct sequencing, it appeared as a novel homozygous in-frame insertion of 9 bp CACTGTGAG at c.3068_3069 position. The identified insertion had a duplication of CACTGT nucleotides at this position. This insertion was also confirmed in second PCR product using forward and reverse sequencing. Such miscall results in Asper analysis have been described previously [16] and thus signifies the limitation of APEX sequencing chemistry for accurate detection of indels. However, it also suggests that the presence of novel indels can be traced if the same lies in the close proximity of the target region. The c.3068_3069insCACTGTGAG is located at the C terminus in guanylyl cyclase domain region of *GUCY2D*. It leads to in-frame insertion of three amino acid residues serine, threonine & valine between 1023 and 1024 codon position; possibly causing a conformational change in the protein structure. A nonsense homozygous *AIPL1* mutation c.834G>A (p.W278X) was found in one patient (LCA69–1). *AIPL1* mutations are grouped into three classes. The class I and II mutations are located in the N-terminus and TPR motifs respectively, and found to be associated with LCA. The class III mutations are small in-frame deletions located in the C-terminus and appear to be associated with autosomal dominant cone rod dystrophy (CRD) and juvenile retinitis pigmentosa (RP) [28]. The AIPL1 (p.W278X) mutation belongs to class II mutation occurring in TPR motifs region. This mutation was described previously, showing a markedly different secondary structure and thermal instability of mutated protein compared to wild type. In addition, unlike wild type which is distributed throughout cytoplasm and nucleus, the W278X mutated protein is located only in the cytoplasm as an aggresome-like particle and gets ubiquitinated indicating its proteosomal degradation [29]. The protein stability tools also support the instability of the mutant (p.W278X) AIPL1 protein. This mutation was reported to be very frequent in Italian cohort of LCA patients [30]. One patient (LCA53–1) carried the mutation in both *CRX* and *IQCB1* genes. The *CRX* mutation c.196 G>A (p.V66I) occurred in the heterozygous state. The mutation is located in the conserved homeo domain of the *CRX* transcription factor. It was previously reported in Spanish early-onset ARRP cohort [17]. In the same patient, another homozygous mutation *IQCB1* c.1465C>T (p.R489X) was observed, which lies in the IQ calmodulin-binding region domain of *IQCB1* with highly conserved nucleotide and moderately conserved amino acid location. This mutation was previously reported in patients with Senior-Loken syndrome [31] and later in LCA patients [32]. A nonsense *RPGRIP1* c.3565 C>T (p.R1189X) mutation was identified in one patient (LCA81–1). The mutation was recently reported in LCA patients by implementing autozygome analysis and exome sequencing in a large cohort of patients with different clinical RD subtypes in Saudi Arabia [33]. An Asper chip variant *RPGRIP1* c.1639 G>T was observed in 5 LCA patients. This variant was initially reported pathogenic in recessive cone-rod dystrophy [34] but other studies [35] including ours, suggest it a polymorphism as it was found in our control group at higher frequency.

## Discussion

In this study, a comprehensive mutation screening of LCA genes emphasizing *RPE65* was performed considering its role in emerging gene therapy. In total, 11 out of 30 (36.6%) cases, revealed ten different pathogenic variations including two novel mutations in *RPE65* and one novel 9bp insertion in *GUCY2D* gene. Segregation of disease alleles were confirmed in available family members carrying the genetic defects. In our study, *RPE65* mutations were found to be the main cause for LCA followed by *GUCY2D*. The previous studies have shown the prevalence of mutations in *RPE65* ranging from 1.7% to 16% in LCA cohort from various geographical origins [24], [36], [37]. We found *RPE65* mutations in 16.6% of the total studied LCA cases, which is higher in contrast to earlier Asian reports showing 1–2% frequency [37], [38]. At c.361 codon position, *RPE65* mutations were found in three cases and there are other mutations previously reported at this site, suggesting it as a mutational hot spot in Indian LCA patients. The *CEP290* mutations particularly c.2991+1655A>G (*p.Cys998X*), which is quite frequent in European cohort of LCA patients, was not observed in South Indian LCA cases. This supports our previous report that mutations that are common in European LCA cohort are not present in Indian population [39].

The clinical evaluation of LCA patients identified with mutations showed a range of primary and secondary features associated with LCA. In all cases, the onset of disease was since birth and all patients were presented with sluggish pupil responses. The ERG recordings in all cases were below the threshold of sensitivity of the ERG system applied. Various types of nystagmus like horizontal jerky, rotatory, pendular and asymmetrical pendular were observed. In one patient (LCA81–1) identified with nonsense *RPGRIP1* mutation, the clinical diagnosis indicated a cornea plana (lesser K readings suggesting a flater cornea) in contrast to keratoconus, an associated clinical feature of LCA described usually with *AIPL1* and *CRB1* mutations. However, this clinical finding may be independent of LCA and need to be confirmed by other evidences. The documented secondary features like photophobia, night blindness and oculodigital sign were appeared in many cases studied, while an alternate exotropia was also observed in one case (LCA 72–1). Axial length of the eye was found in a normal range among all LCA patients. The fundus examination showed features ranging from normal to a pigmented retina with RPE vessel attenuation and mottling. The OCT examination showed the loss of foveal thickness in most of the patients. LCA phenotype in a larger patient group will be required for a compendium of clinical phenotype based on the genotype facilitating prospective diagnosis.

A comprehensive mutation analysis of all LCA genes will involve screening of at least 300 exons in more than 20 genes. Due to huge genetic heterogeneity, large-scale LCA mutational screening is a cumbersome process. Direct sequencing, though a gold standard technique, cannot be applied to screen all LCA genes, as because it would be very expensive, time-consuming and labour intensive. The low-cost molecular tools like single-strand conformational polymorphism (SSCP), denaturing high-performance liquid chromatography (DHPLC), or denaturing gradient gel electrophoresis are not well suited for application of screening numerous genes simultaneously as they remain tedious, time -consuming and no more relevant to date. A more inclusive available method is the customized gene based re-sequencing chip, which is also able to detect novel variants. However, it covers very few LCA genes, with limitation of detecting large heterozygous deletions and duplications, and carries a time-consuming result interpretation [40]. Recent LCA studies have entered into more advanced approaches like genotyping arrays based whole-genome homozygosity mapping and next generation sequencing based whole exome and gene panel analysis [41], [42], [43], [44]. These advanced technologies and APEX are grievously comparable, as the former deals with the major research objectives including novel candidate genes identification, allow discovering new sequence variants, and generate extensive data to be comprehensively analyzed. APEX technology, on the other hand, works with previously known markers, applied as a first-line genetic testing tool to identify only selected known sequence variants and generate simplified data, which can be analysed precisely. APEX method is added with advantages like flexible array format, easy to add new variations and being time and cost-effective. For example, the assay requires time-on-hands as 6 hr, and cost of screening one sample for 15 major LCA genes comes as ‘€ 340’ which is as comparable as other medical diagnostic costs. The APEX platform can be more effective for the diagnosis of inherited disorders with well-defined mutation spectrum.

Many studies have analyzed the patient groups using the Asper mutation chip. The related screening approaches using Asper chip analysis involved testing the samples for known variations and their confirmation with direct sequencing. In some studies, on identification of one mutation, all protein-coding exons of the relevant genes were sequenced [45]. In another approach, patients excluded for mutations in Asper chip analysis were examined for all coding regions of LCA genes by direct sequencing [7]. In other studies, DNA array chip has been used to exclude known LCA mutations among cases in order to apply them for homozygosity mapping or whole exome sequencing analysis [41], [42]. In our study, a consecutive approach was applied, where samples with the absence of mutations in RPE65 in direct sequencing analysis, were subjected to the DNA micro array based Asper chip testing, to look for the presence of mutations in other known LCA genes. This kind of approach is instrumental as it makes availability of complete screening of important gene (s) along with analysis of known vulnerable genetic regions of other related genes in selected negative cases. Thus, it enhances the reliability of primary analysis and productivity of identification of mutations among negative cases. In our analysis, many changes including truncating mutations were found to be frequent among controls and patients. This signifies that such extensive testing can frequently identify a gene with two mutated alleles and in addition, a potentially disease-causing mutation in another retinal disease gene. An another suitable approach for comprehensive screening of LCA genes was used in Chinese population where 51 most frequently mutated exons with their flanking introns in 15 LCA genes were selected for initial screening through direct sequencing [46]. Such approaches combined with Asper chip analysis can be potentially more useful for extensive, highly efficient and cost-effective mutation screening of LCA genes. The population specific screening of LCA genes using such approaches will be helpful in developing an effective tool for molecular diagnosis and studying the gene specific LCA mutational spectrum, which may play a pivotal role for the improvement of emerging gene therapy.
